# Examining mechanisms connecting child maltreatment and interpersonal violence to young adult pain symptoms in women

**DOI:** 10.1097/PR9.0000000000001370

**Published:** 2026-01-09

**Authors:** Sarah J. Beal, Kim Barnett, Susmita Kashikar-Zuck, Chris King, Constance A. Mara, Katie Fox, Mary V. Greiner, Jennie G. Noll

**Affiliations:** a Department of Pediatrics, University of Cincinnati College of Medicine, Cincinnati, OH, USA; b Cincinnati Children's Hospital Medical Center, Cincinnati, OH, USA; c University of Rochester, Rochester, NY, USA; d Mt. Hope Family Center, Rochester, NY, USA

**Keywords:** Child maltreatment, Adult pain, Interpersonal violence, Negative affect

## Abstract

**Introduction:**

Child maltreatment has been linked to elevated pain symptoms and more chronic pain in adults. The hypothalamic–pituitary–adrenal (HPA) axis may be a mechanism connecting maltreatment to pain, where early life stressors are thought to impact HPA axis function, leading to increased inflammation and pain.

**Objectives:**

This study explored pathways associated with pain in young adult women with and without maltreatment exposure. Maltreatment was expected to be associated with increased exposure to interpersonal violence, inflammation, and negative affect which, in turn, would be associated with more pain in adulthood.

**Methods:**

Fifty women randomly selected from a prospective longitudinal cohort (n = 25 with confirmed child maltreatment, n = 25 demographically similar women without history of maltreatment) completed comprehensive assessments of risk factors for pain, including collecting inflammatory biomarkers.

**Results:**

Women who had experienced child maltreatment reported more exposure to interpersonal violence as young adults (χ^2^ [1] = 6.52, *P* = 0.01). Small effect size differences were observed between women with maltreatment exposure and those without for inflammation in young adulthood (*d* = 0.29) and negative affect scores (*d* = 0.47), although not statistically significant. Small-to-moderate effect sizes differences were also observed between women exposed to interpersonal violence and those without interpersonal violence exposure for negative affect (*d* = 0.62) and inflammation (*d* = 0.46). Negative affect was significantly associated with experiencing any pain in young adulthood (odds ratio = 1.12, *P* = 0.03, 95% confidence interval = 1.01, 1.24).

**Conclusion:**

Mechanisms connecting maltreatment to pain are complex. Studies that account for the effect-moderating impact of early life stress on inflammatory pathways alongside psychological effects of stress exposures more proximal to pain (eg, interpersonal violence, mood) are needed.

## Abstract 2

Negative affect, not maltreatment or violence exposure per se, was associated with increased pain in this pilot study of young adult women; larger studies are needed to confirm.

## 1. Introduction

Pain among young adults is increasing^21^ and associated with school and work absenteeism, reduced quality of life, and increased medication use for pain.^24,25^ A significant public health concern,^21^ pain prevention and intervention to reduce the prevalence of chronic pain conditions beginning in adolescence is needed.^1^

The impact of exposure to child maltreatment (ie, abuse or neglect)^19^ on health across the lifespan has increased the attention to the modifiable pathways connecting childhood adversity and adult health.^17,18^ Prospective^4,5,20^ and retrospective^2,6,33^ studies indicate that child maltreatment is associated with more frequent experiences of pain, higher pain intensity, and more chronic pain in adulthood. Child maltreatment and other indicators of adversity are also associated with altered biological and hypothalamus–pituitary–adrenal axis functioning, impacting physiological responses to stress^12,32^ and associated inflammatory pathways^7,11^ that are dysregulated in the context of chronic pain.^9,26^ However, multiple intervening factors (eg, exposure to violence, psychological well-being, health behaviors)^13,15^ may also contribute to increased pain in adults,^14,16^ including those who were exposed to child maltreatment. Understanding the factors associated with pain is important for guiding future research and prevention efforts that will minimize the impact of maltreatment on adult outcomes.

This pilot study examined the association of maltreatment with exposure to interpersonal violence, inflammation, and psychological well-being as factors associated with pain and potential pathways by which maltreatment impacts pain experiences in adulthood. It was expected that maltreatment (past) and interpersonal violence exposure (current) would be associated with increased inflammation (C-reactive protein [CRP]), and worse psychological well-being (negative affect), which would, in turn, be associated with increased pain in young adulthood.

## 2. Methods

### 2.1. Participants and procedures

Fifty women (M = 28.6 years, SD = 1.5, range 26.21–32.33 years; 46% non-Hispanic White, 42% Black) who enrolled in a prospective longitudinal study when they were aged 12 to 17 years^22,23^ and participated in a follow-up survey, as young adults (87% retention)^4^ were selected at random (25 with maltreatment, 25 without) for the current study. Randomly selected participants who declined (N = 13) or did not respond (N = 124) were replaced with a new random selection until 25 participants were enrolled in each group (26% participation). Recruitment occurred between August 2021 and April 2022. The enrolled and potentially eligible samples did not demographically differ (*P*s > 0.10).

Participants completed surveys through REDCap that assessed interpersonal violence exposure,^31^ positive and negative affect over the past week,^35^ and pain experiences.^10^ A fasting blood draw was collected during study visits to measure CRP,^29^ and a trauma narrative was completed.^27^ Study participants were compensated up to $135. The Institutional Review Board at Cincinnati Children's Hospital approved the study.

### 2.2. Measures

Maltreatment exposure (0 = no, 1 = yes) was determined in the parent study.^22,23^ Interpersonal violence exposure (0 = no, 1 = yes) used items from the Revised Conflict Tactics Scale (“My partner pushed, shoved, or slapped me,” “My partner punched or kicked or beat me up”) or report of interpersonal violence after the age of 18 years.^27^ Negative affect was measured using the subscale from the PANAS.^35^ Serum CRP was assayed by the clinical research laboratory at Cincinnati Children's. Any pain (0 = no, 1 = yes) was measured using the item “Throughout our lives, most of us have had pain from time to time (such as minor headaches, sprains, and toothaches). Have you had any pain other than these types in the last week?^10^

### 2.3. Analysis plan

Descriptive and bivariate statistics examined demographic and clinical variables by maltreatment status and exposure to interpersonal violence. Group differences were examined using Welch *t*-tests, and standardized effect size estimates (Cohen d) were computed (small: *d =* 0.20–0.49, medium: *d =* 0.50–0.79, large: *d* ≥ 0.80).^8^

Three models were estimated using full information maximum likelihood estimation and a robust variance estimator: (1) linear regression of the effects of maltreatment and interpersonal violence status (predictors) on negative affect (outcome), (2) linear regression of the effects of maltreatment exposure and interpersonal violence (predictors) on log-transformed CRP (outcome), and (3) logistic regression of the effects of maltreatment, interpersonal violence, affect, and CRP (predictors) on pain (outcome). Before conducting inferential analyses, assumptions of normality, linearity, homoscedasticity, and model fit were assessed for all continuous outcomes. C-reactive protein was positively skewed; values were log-transformed before analysis. As a sensitivity check, models were rerun excluding 3 participants with CRP values >2 SD above the mean. All other model assumptions were met. Analyses were conducted in Stata version 18^30^ with alpha set to 0.05. A priori power analyses indicated that the target sample size would provide 80% power to detect an incident rate difference of approximately 0.38 for binary outcome and a standardized mean difference of d ∼0.8 for continuous outcomes, assuming α = 0.05 and β = 0.2.

## 3. Results

### 3.1. History of maltreatment

Most (72%) young women who experienced maltreatment reported exposure to interpersonal violence as adults, significantly higher than for women who were not exposed to maltreatment (36%; Table 1). In regression models, maltreatment was not statistically significantly associated with CRP (b = 0.23, *P* = 0.60, 95% CI [confidence interval] = −0.62, 1.07) with a small effect size difference (*d* = 0.29). Maltreatment was also not associated with negative affect (b = 2.11, *P* = 0.47, 95% CI = −3.65, 7.87); a small effect size difference was observed (*d =* 0.47).

**Table 1:** Descriptive statistics and bivariate comparisons for maltreatment exposure, interpersonal violence exposure, C-reactive protein, and negative affect.

	Maltreated women (N = 25)	Non-maltreated women (N = 25)	Bivariate comparison
Exposure to interpersonal violence in adulthood (IPV), N (%)	18 (72)	9 (36)	χ^2^ (1) = 6.52, *P* = 0.01
Log-transformed CRP mean (SD)	8.70 (12.13)	3.83 (4.25)	t (23.3) = −1.71, *P* = 0.10
Negative affect mean (SD)	23.48 (8.32)	19.64 (8.15)	t (47.48) = −1.61, *P* = 0.11

	Women with IPV (N = 27)	Women without IPV (N = 23)	Bivariate comparison
Log-transformed CRP mean (SD)	7.97 (11.46)	3.94 (4.48)	t (29.75) = −1.56, *P* = 0.13
Negative affect mean (SD)	26.67 (8.47)	18.67 (7.53)	t (47.18) = −2.16, *P* = 0.04

### 3.2. Current exposure to interpersonal violence

The regression models indicated no statistically significant association between interpersonal violence and CRP (b = 0.60, *P* = 0.19, 95% CI = −0.27, 1.37), although a small effect size difference was observed (*d =* 0.46) (Table 1). Similarly, interpersonal violence was not statistically significantly associated with negative affect (b = 4.10, *P* = 0.16, 95% CI = −1.57, 9.76), with a moderate effect size difference (*d =* 0.62).

### 3.3. Potential pathways linking maltreatment and pain

Logistic regression with maltreatment status, interpersonal violence exposure, log-transformed CRP, and negative affect scores as predictors of reporting any pain found that worse negative affect was associated with significantly higher odds of reporting any pain (odds ratio [OR] = 1.12, *P* = 0.03, 95% CI = 1.01, 1.24). Higher CRP was associated with lower odds of reporting any pain (OR = 0.93, *P* = 0.03, 95% CI = 0.86, 0.99).

Post hoc analyses identified 3 participants with log-transformed CRP values >2 SD above the mean. When these participants were excluded from the logistic regression model, the association between CRP and pain was no longer statistically significant, and the effect size was very small (OR = 1.03, *P* = 0.67, 95% CI = 0.89, 1.19). Linear regression models predicting CRP and negative affect remained nonsignificant and in the same direction when reestimated.

## 4. Discussion

This study examined associations among adult interpersonal violence exposure, inflammation, affect, and pain in young adult women with and without a confirmed history of child maltreatment exposure. We proposed that child maltreatment and interpersonal violence would be associated with dysregulated inflammatory pathways (elevated CRP) and higher negative affect, leading to increased pain. There were small to moderate effect size differences in inflammation (CRP) and negative affect that, although not statistically significant, suggest that child maltreatment and interpersonal violence may contribute to increased inflammation and worse mood. Although there was no direct effect of maltreatment or interpersonal violence on pain, negative affect was significantly associated with increased pain in young adulthood.

This study prospectively established cohorts with and without child maltreatment, confirmed with trauma interviews to minimize contamination in the nonmaltreated sample.^3,28^ Adult exposure to violence that might confound the relationship between child maltreatment and adult outcomes was accounted for.^34^ The inclusion of historical exposure to maltreatment and current exposure to violence allowed us to examine unique pathways connecting adverse experiences to inflammation and affect, highlighting the complex mechanisms that may lead to elevated risk for pain in adulthood.^9,20^

Maltreatment and violence exposure did not directly predict pain in this study, which could occur for several reasons. First, wear and tear of any violence exposure (in childhood or adulthood) may take time to manifest, such that in this sample, more time may be needed to see the effects of maltreatment on inflammation on pain. This is consistent with a recent meta-analysis suggesting a weak association between child maltreatment and chronic pain in middle adulthood.^20^ Second, it may be that the timing of exposure to adversity over the lifespan matters most, where exposure earlier in childhood (before adolescence, as observed in this study) uniquely impacts inflammatory pathways in a way that exposure at other periods in the lifespan (eg, adulthood) does not.^9^ This could help to explain the disparate findings regarding maltreatment and pain in the literature.^20^

This study is limited by small sample sizes and cross-sectional assessments of CRP (which indicates an inflammatory process, not necessarily chronic inflammation),^29^ mood, and pain. Participation rates were low, which may have contributed to a nonrepresentative sample. A single item assessing pain is an additional limitation. Replication with larger sample sizes (eg, 180 participants for *d* = 0.29) may result in the detection of moderate effect size differences as statistically significant. The association between greater negative affect and increased odds of reporting any pain reached statistical significance with a modest effect size (OR = 1.12) with relatively narrow confidence intervals (95% CI: 1.01, 1.24). This may reflect a chance finding or sampling variability or may be due to the binary outcome for the single item indicating any pain in the past week. Although this finding should be interpreted cautiously, it may reflect a true but modest association that warrants replication in larger, better-powered studies. Despite these limitations, this pilot study generates new questions about how maltreatment and other stressful life events may, over time, contribute to the onset of pain in adulthood—negative affect, for example, is modifiable and could be leveraged to prevent pain.

## Disclosures

The authors have no conflict of interest to declare.
